# Clinical efficacy and potential mechanisms of acupuncture for Parkinson’s disease: the role of GABAergic signaling

**DOI:** 10.3389/fnins.2025.1525486

**Published:** 2025-02-19

**Authors:** Wenhui Lu, Ting Zhang, Minghui Li, Jun Zhang, Ningning Liu, Lanfang Yang, Guomin Huang

**Affiliations:** ^1^Affiliated Rehabilitation Hospital of Nanchang University, Nanchang, Jiangxi, China; ^2^The First Affiliated Hospital of Nanchang University, Nanchang, Jiangxi, China; ^3^Affiliated Hospital of Jiangxi University of Chinese Medicine, Nanchang, Jiangxi, China

**Keywords:** acupuncture, Parkinson’s disease, GABAergic signaling, neurological disorders, clinical efficacy and mechanisms

## Abstract

Parkinson’s disease (PD), a common neurodegenerative disease, seriously impacts the quality of life of patients. In recent years, research has revealed that the GABAergic signaling pathway plays an important role in the occurrence and development of PD. GABA is an important inhibitory neurotransmitter in the central nervous system (CNS), synthesized through the alpha decarboxylation reaction of glutamate (Glu) under the action of glutamic acid decarboxylase (GAD) in GABAergic neurons. It works by binding to specific receptors (GABA_*A*_/GABA_*B*_). In PD patients, the activity of GABAergic neurons in the basal ganglia (BG) changes, leading to an imbalance between direct and indirect pathways and causing motor symptoms. Meanwhile, the GABAergic signaling pathway is closely related to non-motor symptoms such as anxiety, depression, and sleep disorders. At present, the treatment methods for PD mainly include drug therapy, surgical treatment, and rehabilitation therapy. Acupuncture, as a complementary and alternative therapy, has shown promising efficacy in the clinical treatment of PD. This review comprehensively and thoroughly explores the therapeutic effect of acupuncture on PD and its mechanism of action with GABAergic signaling. By systematically summarizing relevant research results, it was found that acupuncture may exert a complex mechanism of therapeutic effect by regulating the GABAergic signaling pathway. Further clarification of these mechanisms of action will be beneficial for promoting the application and development of acupuncture in the treatment of PD and is expected to provide new targets and strategies.

## 1 Introduction

Parkinson’s disease (PD) is a neurodegenerative disorder characterized by motor symptoms such as tremors, muscle rigidity, and bradykinesia, as well as non-motor symptoms such as anxiety, depression, and sleep disorders (Kalia and Lang, 2015). PD is the second most common neurodegenerative disease in the elderly population. With the global population aging accelerating, the risk of developing PD has also risen. In addition, with the improvement of living standards, environmental changes such as heavy metals, industrial chemicals, air pollution, as well as high-fat diets and lack of exercise, may also increase the risk of PD (Ben-Shlomo et al., 2024). PD is an increasing challenge to public health. Some studies, analyzing data from 1990 to 2019, have found that the burden of PD in the world and most regions and countries showed an upward trend, including the incidence rate, prevalence and overall years lived with disability (YLDs) (GBD 2016 Neurology Collaborators, 2019; Ou et al., 2021). As a chronic progressive disease, PD requires long-term medical care and medication treatment. At the same time, as the condition progresses, patients may also experience symptoms such as pain, motor dysfunction, swallowing difficulties, and urinary incontinence, which imposes a heavy burden on their families and society (Fasano et al., 2015; Denmon et al., 2023).

The control and management of PD are urgently needed. At present, the treatment of PD mainly includes drug therapy, surgical therapy, and rehabilitation therapy, but these treatment approaches have certain limitations. As one of the oldest and widely used technologies in the world, acupuncture has been recommended by the World Health Organization (WHO) as a strategy to improve a variety of diseases and symptoms (World Health Organization, 2003). In recent years, as a traditional Chinese medicine therapy, acupuncture has shown advantages in the treatment of PD and the potential therapeutic mechanisms have gradually attracted attention (Fan et al., 2022). An increasing number of studies have shown that acupuncture may improve the symptoms of PD patients by regulating the function of the nervous system (Zhao et al., 2021). The pathogenesis of PD is complex and involves the interaction of multiple factors. Previous studies have involved factors such as degeneration of dopaminergic neurons in the substantia nigra pars compacta (SNpc), abnormal protein aggregation, and neuroinflammation, etc (Raza et al., 2019). Modern research has found that the degeneration or functional abnormalities of γ-aminobutyric acid (GABA) ergic neurons may also play an important role in the pathogenesis of PD, further exacerbating the symptoms and progression of the disease (Sanjari Moghaddam et al., 2017). GABAergic neurons are widely distributed in the brain, and the GABAergic signaling pathway plays an important role in the pathogenesis of PD, thus acupuncture may play a therapeutic role in PD by regulating the function of GABAergic neurons and related signaling pathways. In view of the demand for effective management and treatment strategies for PD, we thoroughly reviewed the basic and clinical research of acupuncture for PD and explored its possible mechanism. This is aimed at providing new evidence for its clinical application and suggesting a promising direction for future research.

## 2 GABAergic signaling in the occurrence and progression of PD

The neurotransmitters in the nervous system can be classified into inhibitory neurotransmitters and excitatory neurotransmitters, which play a crucial role in the functioning of the nervous system (Amantea and Bagetta, 2017). Glutamate (Glu) and acetylcholine (ACh) are the main primary excitatory neurotransmitters in the nervous system. GABA is the most important inhibitory neurotransmitter in the central nervous system (Roberts, 1986). GABAergic neurons refer to neurons that can synthesize and release the inhibitory neurotransmitter GABA. GABAergic neurons exert a crucial inhibitory regulatory function in the nervous system by binding to specific receptors, reducing neuronal excitability, and maintaining functional balance of the nervous system (Sanjari Moghaddam et al., 2017). For a long time, neurons have been considered the only cell type in the nervous system capable of releasing neurotransmitters for information transmission. However, with the continuous deepening of research, it has been discovered that except neurons, some other glial cells, especially astrocytes, can also release neuroactive substances such as glutamate, GABA, ATP (Lei et al., 2024). These substances can further act on neurons or other glial cells, and this process is called gliotransmission (Lee et al., 2010; Pirttimaki et al., 2017). Gliotransmission may also be involved in the pathogenesis of PD. A study has found that a large number of tyrosine hydroxylase (TH)-negative/ DOPA decarboxylase (DDC)-positive dormant neurons were detected in SNpc of autopsy PD patients, surrounded by a large number of GABA positive astrocytes (Heo et al., 2020). This finding diverges from traditional views and offers novel perspectives on the pathogenesis and treatment of PD.

γ-aminobutyric acid is formed by the alpha decarboxylation reaction of glutamate under the action of glutamic acid decarboxylase (GAD, consists of two major isoforms with 65 and 67 kD differing molecular weights) in GABAergic neurons, and further metabolized into succinic acid under the action of GABA transaminase (GABA-T) and succinate semialdehyde dehydrogenase (SSADH). Glu is converted from excitatory neurotransmitter to the inhibitory neurotransmitter GABA under the catalysis of GAD (Martin, 1993; Martin and Rimvall, 1993). This transformation process plays an important role in maintaining the balance between excitation and inhibition in the nervous system (Petroff, 2002). The generated GABA is stored in the synaptic vesicles via vesicular GABA transporter (VGAT) of GABAergic neurons and released into the synaptic cleft when neurons are excited (Agner, 2001). When the GABAergic neurons are excited, action potentials propagate along axons to presynaptic terminals. This leads to the opening of Ca^2+^ channels and the influx of Ca^2+^ into the presynaptic terminals. The influx of Ca^2+^ is a key signal that triggers the release of GABA. Meanwhile, studies have found that glutamatergic neurons can express VGAT (1,2,3) and GABA-T/SSADH. After GABA is released, it can be reabsorbed by glutamatergic neurons and recycled to form Glu. GABAergic neurons can also absorb extracellular Glu, re-stimulate GAD, promote GABA synthesis, and thus increase GABA levels (Roettger and Amara, 1999; Bak et al., 2006). There are several different mechanisms of GABA release from astrocytes. The release of GABA mediated by astrocytes has been controversial, as the expression levels of the main GABA synthase glutamate decarboxylase in astrocytes are relatively low and may not be sufficient to support non-vesicular GABA release (Lei et al., 2016). However, recent studies have shown that GABA in astrocytes can be released from astrocytes through GABA-permeable anion channels or via GABA transporters, at least in certain regions of the CNS (Kalia and Lang, 2015). For example, Yang J. et al. (2023) found that astrocytes in ventral tegmental area (VTA) can release GABA through the volume-regulated anion channel Swell1 (also known as *Lrrc8a*, Leucine-rich repeat containing family 8a) to regulate the activity of GABA neurons and modulate cocaine-induced locomotor and reward behaviors in mice. In addition, GABA-permeable Bestrophin-1 (Best1) channel also play an important role in the release of GABA from astrocytes (Lee et al., 2010; Pandit et al., 2020). GAT-mediated GABA released from astrocytes may another important way. The expression of GAT-1 and GAT-3 is brain-specific, where GAT-1 protein is both in neurons and astrocytes, and GAT-3 is preferentially localized on the processes of astrocytes (Minelli et al., 1995, 1996). Thus, GAT-3 is widely believed to contribute to the release of GABA from astrocytes in the brain. A study has found that when the nervous system is damaged, astrocytes, rather than neurons, exhibited a transient depolarization, which promotes non-synaptic GABA release via GAT-3. When using a blocker of the GABA transporter GAT-3 (SNAP5114) in astrocytes, GAT-3 operates in a release mode in wild-type mice. The application of transportable glutamate transporter substrate (D-aspartate) can restore the release of non-synaptic GABA in astrocytes of mice (Wójtowicz et al., 2013).

γ-aminobutyric acid exerts its effect by binding to specific receptors on neurons. These receptors are mainly divided into two types, responsible for rapid and slow inhibition of neurotransmission, including GABA_*A*_ receptors and GABA_*B*_ receptors (Sharma et al., 2023). GABA_*A*_ is a ligand gated Cl^–^ channel receptor. When GABA binds to the GABA_*A*_ receptor, GABA_*A*_ opens the Cl^–^ channel, allowing Cl^–^ to flow in, while reducing the resting potential of the cell membrane and producing inhibitory 0 in the brain (Weir et al., 2017). GABA_*B*_ is a metabolic receptor that belongs to the G protein coupled receptors. After GABA binds to the GABA_*B*_ receptor, it can activate G protein, regulate K^+^ and Ca^+^ channels by increasing K^+^ levels and preventing Ca^+^ release, further inhibit the release of other neurotransmitters into the presynaptic membrane (Blein et al., 2000). Modern research has found that various neurodegenerative diseases are associated with low levels of GABA, including PD. Further studying the mechanisms of GABA receptors to explore new targets and therapeutic pathways, is of great significance for effective management of GABA related PD (Blein et al., 2000).

### 2.1 Motor dysfunction

The basal ganglia (BG), consisting of striatum (STR), pallidum, substantia nigra (SN), and subthalamic nucleus (STN), is a key brain area involved in motor regulation, and its function is abnormal in PD (Calabresi et al., 2014). GABAergic neurons are widely distributed in the basal ganglia and participate in sensory perception. When GABA is deficient, it can lead to dyskinesia or bradykinesia in PD patients. When GABAergic neurons are excessively excited, it may cause excessive muscle tension, resulting in muscle stiffness. A post-mortem study observed the expression of inhibitory and excitatory neurotransmission (dopaminergic, GABAergic, and glutamatergic neurons) in the basal ganglia thalamocortical circuit of Parkinson’s syndrome patients. It was found that in Parkinson’s disease patients, GABA levels in the central medial thalamus were reduced by 36% compared to deceased individuals without any history of neurological or psychiatric disorders or neuropathological abnormalities (Gerlach et al., 1996). PD can be divided into postural instability gait difficulty (PIGD) and tremor-dominant (TD) subtypes. Some studies have also evaluated the differences in GABA levels between PD motor subtypes and found that compared with the healthy individuals, the GABA levels in the left BG area of PD patients were significantly reduced. And in the TD group, the concentration of GABA was lower than that in the PIGD group (Gong et al., 2018). In addition, a GABAergic deficit in the brainstem may also lead to PD (Song et al., 2021). Experimental studies also have shown that the dysregulation of GABAergic neurons in the substantia nigra pars reticulata (SNr) leads to abnormal firing frequency and pattern of neurons in a rat model of PD (Wang et al., 2010). In PD patients, the expression of GABA receptors also changes. For example, a study has found that reduced GABA_*A*_ receptor availability in the thalamus is associated with worsening axial motor impairments in PD, but not with nigrostriatal degeneration (Bohnen et al., 2023). When the inhibition from GABA in the BG and other areas decreases, the activity of these motor regulatory nuclei increases, further exacerbating the impact on basal ganglia output and leading to worsening of motor symptoms.

### 2.2 Anxiety and depression

Parkinson’s disease patients often have emotional disorders such as anxiety and depression (Ray and Agarwal, 2020). Anxiety and depression in PD patients are associated with changes in brain structural connectivity (Carey et al., 2023). Some areas of the brain, such as the amygdala, prefrontal cortex, dorsal raphe nucleus (DRN), hippocampus and anteroventral bed nucleus of stria terminalis (avBNST), are closely related to emotions, and these areas also have a large number of GABAergic neurons (Lydiard, 2003). GABA may be closely related to anxiety and depression. An increasing amount of preclinical and clinical evidence emphasizes the impact of changes in gut microbiota on emotions. Research has found that the gut microbiota plays an important role in anxiety-like behavior by altering the function of the GABA system (Yao et al., 2022). Oligodendrocyte precursor cells (OPCs) have the ability to directly sense neuronal synaptic inputs. When stimulated, they can drive GABA release and enhance inhibitory synaptic transmission in neurons, triggering anxiety-like behavior in the body (Zhang et al., 2021). In PD patients, some GABAergic neurons in the brain related to emotion regulation are impaired, resulting in reduced release or weakened effects of GABA, resulting in insufficient inhibition of the nervous system and relatively increased neuronal excitability, making it difficult for patients to effectively cope with negative emotions. A study has found that unilateral 6-hydroxydopamine (6-OHDA) injury in the substantia nigra pars compacta (SNc) of rats induces anxiety-like behavior, accompanied by a decrease in DA levels in the basolateral amygdala (BLA), which may be related to impaired GABAergic neuron function. Further investigation revealed that the synthesis and release of GABA increase, and the expression of GABA_*A*_ receptor subunits is upregulated in avBNST. Activation and blockade of avBNST GABA_*A*_ receptors may affect the firing activity of VTA dopaminergic and DRN serotonergic neurons, thereby regulating the release of dopamine (DA) and 5-HT in BLA (Li R. et al., 2023). Chemogenetic activation of avBNST*^GABA^*-VTA or avBNST*^GABA^*-DRN pathway also induced anxiety-like behavior in PD rats and reduced the release of DA or 5-HT in BLA (Li R. et al., 2024).

### 2.3 Cognitive impairment

Parkinson’s disease is the second most common neurodegenerative disorder, and cognitive impairment is also common among various non-motor symptoms that can occur at any stage of PD (Aarsland et al., 2021). Clinical studies have indicated that early PD patients exhibit visual spatial/executive, memory, attention, and language impairments, etc (Mills et al., 2020). GABAergic neurons are involved in regulating processes such as memory and learning. For example, abnormal GABAergic neurons in the hippocampus may affect the formation and consolidation of memory. Rats induced by middle cerebral artery occlusion with high hippocampal GABA levels may display poorer cognitive outcomes (Torres-López et al., 2023). The cognitive decline in PD patients may be related to changes in GABAergic signaling in the brain. A study has shown that there is a linear correlation between the levels of GABA/glutamate-glutamine (Glx), Stroop Word-Color Test (SWCT) time/error interference effects and Wisconsin Card Sorting Test (WCST) perseverative errors in the cerebellum of PD patients, and they have different impacts on cognitive markers of PD (Piras et al., 2020). The level of GABA is also crucial for accuracy and discrimination. A cohort study found that a decrease in GABA levels is associated with visual hallucinations in PD patients (Firbank et al., 2018). Retinal imaging shows that α-synuclein (α-syn) accumulates in the retinal ganglion cell layer and arterial vascular edges of PD mice. The accumulation of α-syn persists and increases with age, leading to cognitive impairment (Price et al., 2016).

### 2.4 Sleep disorders

Sleep disorders are common in Parkinson’s disease, including insomnia, daytime sleepiness, REM sleep behavior disorders, sleepwalking, overlapping sleep disorders, restless leg syndrome (Stefani and Högl, 2020). Researchers have used smartwatch sensors to monitor significant differences in the percentage of REM abnormalities and deep sleep stages between PD patients and healthy individuals (Ko et al., 2022). The regulation of sleep involves multiple brain regions, some of which contain GABAergic neurons that play important roles in initiating and maintaining sleep. In PD, sleep disorders may be associated with abnormal GABAergic signaling (Al-Kuraishy et al., 2024). A study has detected serum levels of adenosine, glial-derived neurotrophic factor (GDNF), and GABA, and used logistic regression to explore the correlation between these factors and sleep disorders. PD patients with sleep disorders were found to have higher 24-item Hamilton Depression Scale (HAMD), 14-item Hamilton Anxiety Scale (HAMA), Epworth Sleepiness Scale (ESS), Movement Disorder Society-Unified Parkinson’s Disease Rating Scale III (MDS-UPDRS III) and Hoehn-Yahr (H&Y) staging scores, accompanied by lower levels of adenosine, GDNF, and GABA (Wang et al., 2023). Another study measured the plasma levels of aspartate (Asp), Glu, glycine (Gly) and GABA in 92 PD patients and 60 healthy individuals. It was found that plasma levels of Asp and Glu were negatively correlated with the severity of depression and sleep disorders in PD patients. The decrease in GABA plasma levels is associated with more severe symptoms of sleep disorders. After controlling for gender, disease duration, severity of motor symptoms, and anti-PD medication, this negative correlation still exists (Tong et al., 2015). There are potential opposite changes in hippocampal and reticulo-thalamic (RT) GABAergic parvalbumin (PV) interneurons, as well as differential expression of MAP2 and postsynaptic density protein-95 (PSD-95), which may form the basis of precursor local sleep disorders in the rat models of PD (Radovanovic et al., 2021). These findings above may indicate new GABAergic methods for treating both motor and non-motor impairments in PD.

### 2.5 Discussion

From the above, the GABAergic signaling pathway is involved in the occurrence and development of PD, whether it is motor symptoms or some non-motor symptoms such as anxiety, depression, cognitive impairment, and sleep disorders, etc. Abnormal changes in GABA or GABA receptors expression have been observed in both clinical and basic experiments among these disorders. Further discussion on the mechanism of GABAergic signaling pathway involvement in PD may be related to the following aspects.

Pathologically, PD is characterized by the loss and degeneration of dopaminergic neurons in the SN. Dopaminergic neurons produce a large amount of reactive oxygen species during the synthesis and metabolism of DA. Long-term oxidative stress can lead to mitochondrial dysfunction and insufficient energy supply, resulting in the death of dopaminergic neurons, a decrease in dopamine levels in the striatum, leading to motor symptoms (Simon et al., 2020). There is a direct synaptic connection between GABAergic neurons and dopaminergic neurons. As an inhibitory neurotransmitter, GABA can exert inhibitory regulation on the activity of dopaminergic neurons by releasing it into the synaptic cleft and binding to GABA receptors on dopaminergic neurons. However, when the function of GABAergic neurons is abnormal, such as disorders in the synthesis, release, or reuptake process, the inhibitory effect of GABA on dopaminergic neurons is enhanced or weakened, leading to neuronal degeneration (Brill-Weil et al., 2024). Chronic nasal administration of Kisspeptin-54 can augment the decline in GABA levels in the amygdala induced by 6-hydroxydopamine (OHDA), and effectively mitigated motor deficits and the loss of nigral dopaminergic neurons (Sinen et al., 2024). Oxytocin can inhibit excitatory synaptic inputs onto DA neurons by activating oxytocin receptor-expressed SN GABA neurons, which target GABA_*B*_ receptors expressed in SN DA neurons that project glutamatergic axons. This reduces reduce excitotoxicity and improve 1-methyl-4-phenyl-1,2,3,6-tetrahydropyridine (MPTP)-induced symptoms in PD mice (Wang M. et al., 2024).

**FIGURE 1:**
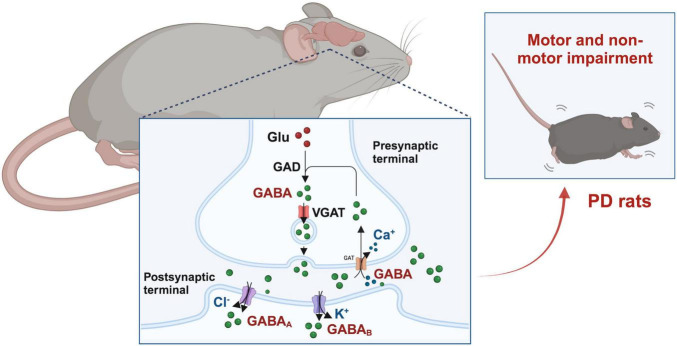
GABAergic signaling in the occurrence and progression of Parkinson’s disease (PD). Glutamic acid decarboxylase (GAD) converts glutamate (Glu) into γ-aminobutyric acid (GABA), which is then stored in synaptic vesicles. GABA is released during presynaptic activation of GABAergic neurons and enters through activation of GABA receptors, leading to postsynaptic membrane hyperpolarization and inhibition. Excess GABA in the synaptic cleft can also be reabsorbed to presynaptic neurons through GABA transporters.

In addition to the mechanism of dopaminergic neuron damage, there is another key factor that affects the progression of PD: neuroinflammation. Microglia are immune cells in the CNS. In the brains of PD patients, microglia are activated and release large amounts of inflammatory factors. These inflammatory factors can cause damage to neurons and promote the development of PD. Astrocytes are also involved in the neuroinflammatory response of PD (Blandini, 2013). Activated astrocytes release some neurotoxic substances, which can also affect the nutrient supply and metabolism of neurons, exacerbating neuronal damage. It is reported that astrocytes in striatum can regulate dopamine transmission by regulating the extracellular levels of GABA and inhibit striatal DA release through GABA_*A/B*_ receptors (Stedehouder et al., 2024). The depolarization of astrocytic mitochondria within the SNc can lead to the accumulation of GABA and glutamate in SNc, subsequently resulting in excitation/inhibition imbalance and motor deficits (Li S. M. et al., 2024). Blocking excessive astrocytic GABA could be an effective therapeutic strategy against PD (Heo et al., 2020). The use of GABA_*A*_ antagonists can also effectively reduce the activation of microglia and astrocytes, increase the expression level of tyrosine hydroxylase (TH), and inhibit α-syn levels, finally improve motor incoordination and impaired locomotor gait, fatigue, anxiety, depression, and impaired short-term memory (Izquierdo-Altarejos et al., 2024). In addition, long-term exposure to environmental toxins may increase the risk of PD, such as per-and polyfluoroalkyl substances (PFAS), etc. A study compared the proteomic dataset between flies exposed to PFAS and a PD fly model expressing human α-synuclein. And it was found that PFAS may regulate GABA-related pathways by modulating astrocytes in the brain, leading to mitochondrial dysfunction and neuroinflammatory responses, resulting in motor deficits (Abu-Salah et al., 2024). In summary, the pathogenesis of PD is complex, and GABAergic signaling may play an important role in it, which can provide new targets and strategies for the treatment of PD. However, the specific mechanism still needs further investigation in the future (Figure 1).

## 3 Clinical efficacy of acupuncture in PD

Acupuncture is one of the most widely used techniques in traditional Chinese medicine. It refers to the use of manual acupuncture (MA), electroacupuncture (EA), acupressure, and transcutaneous acupoint electrical stimulation (TAES) to act on specific acupoints, thereby adjusting the qi and blood of the body’s organs through meridians to treat diseases. Many clinical trials have shown that acupuncture has a significant therapeutic effect on PD patients, which can reduce motor and non-motor symptoms (Table 1; Lee et al., 2008).

**TABLE 1:** Clinical effects of acupuncture for Parkinson’s disease (PD).

References	Participants and methods	Intervention methods	Acupoints	Acupuncture parameters	Effect measures	Outcomes
Lei et al., 2016	A total of 15 participants of PD with gait disorder	EA	GV20, GV14, LI4, ST36, GB34, BL14, SP36, KI13, LR3	4/100 Hz, 100 μS, 3 weeks	STHW, DTHW, STFW, DTFW, UPDRS, SF-12 health survey, FES-I, VAS	STHW↑, STFW↑, DTFW↑, DTFW↑, STFW↑, UPDRS↓, gait speed↑
Toosizadeh et al., 2015	A total of 15 participants of PD with gait disorder	EA	GV20, GV14, ST36, LI4, GB34, LR3, KI3, SP6, BL40	Once a week, 3 weeks	COGML/AP sway, ankle/hip sway, UPDRS, VAS, SHORT FES-I, SF-12, MMSE	COGML/AP sway↓, ankle/hip sway↑, UPDRS↓
Jang et al., 2020	A total of 26 participants of PD with gait disorder	MA	–	Two days per week, 4 weeks	UPDRS	UPDRS↓, stride↑, swing↑, single support times↑, oxyhemoglobin↑
Shaosong et al., 2024	A total of 60 participants of PD with skeletal muscle pain	EA	GV20, CV6, LU7, SI19, LI15, upper pain add LI11, lower pain add SP10, ST36	Once a day, five times from Monday to Friday, 4 weeks	Young’s modulus of biceps, quadriceps and shear wave velocity of biceps, KPPS, VAS, UPDRSII, UPDRSIII, modified Ashworth, HAMD, MAS	Young’s modulus of biceps↓, quadriceps and shear wave velocity of biceps↓, KPPS↓, VAS↓, UPDRSII↓, UPDRSIII↓, HAMD↓, MAS↓
Wang M. et al., 2024	A total of 60 participants of PD with chronic pain	Fire needling therapy	GV16, GB20, UB10, GB12, the ashi point	Each selected point was pricked twice, three times per week, 8 weeks	KPPS, VAS, UPDRS, PDQ-39	KPPS↓, VAS↓, UPDRS↓, PDQ-39↓
Fukuda et al., 2016	A total of 13 participants of PD with dysphagia	MA	ST36, SP6, LR3, LI4, LI11, GB20, BL18, BL23	10–15 min, once	Tongue pressure, swallowing reflex latency, saliva swallow	Tongue pressure↑, mean swallowing reflex latency↑, saliva swallow↑
Fan et al., 2022	A total of 70 participants of PD with anxiety	MA	GV24, GV29, HT7, SP6, Sishenzhen (GV21, GV19, 1.5 cun next to GV20)	180–200 rpm, three times per week, 8 weeks	HAM-A, UPDRS, PDQ-39	HAM-A↑, PDQ-39↑, ACTH↑
Aroxa et al., 2017	A total of 22 participants of PD with sleep disorder	MA	LR3, SP6, LI4, TE5, HT7, PC6	Eight weekly sessions, 8 weeks	HY scale, MMSE, PDSS	PDSS↑
Yan et al., 2024	A total of 83 participants of PD with sleep disorder	MA	GV24, GV29, LI4, LR3, SP6, HT7, ST36, BL62, KI6, Sishenzhen	Three times per week, 4 weeks	PDSS, UPDRS, NMSS, ESS, HAM-A, PDQ-39	PDSS↑, UPDRS↓, NMSS↓, ESS↓, HAM-A↓, PDQ-39↓
Kong et al., 2018	A total of 40 participants of PD with fatigue	MA	PC6, LI4, ST36, SP6, KI3, CV6	Twice-weekly sessions at least 3 days apart, 5 weeks	MFI-GF, MFI-Total score, UPDRS-motor, ESS, GDS, PDQ-39	MFI-GF↓, MFI-total score↓, UPDRS-motor↓
Kluger et al., 2016	A total of 94 participants of PD with fatigue	MA	GV20, GV24, LI10, HT7, ST36, SP6	Twisted three times to the right, every 2, 6 weeks	MFI, PDQ-39, HADS, ESS↓, AES	MFI↓, PDQ-39↓, HADS↓, ESS↓, AES↓
Nazarova et al., 2022	A total of 30 participants of PD with sleep disorder and fatigue	Scalp-abdominal EA	GV20, GB20, SP6, SP6, ST25, LI4, LR3, ST40	Twice a week, 8 weeks	NMSS, PDSS, BSFS, PAC-QOL, UPDRS, HY scale	NMSS↓, PDSS↑, UPDRS↓
Eng et al., 2006	A total of 25 participants of idiopathic PD	Tui na+MA+qigong	ST42, SP3, LI11, LI15, LI20, ST7, ST36	8/14 Hz, once a week, 6 months	UPDRS, UPDRS, PDQ-39, BDI	UPDRS (I/II)↓, UPDRS III↓, PDQ-39↓, BDI↓

↑, Upregulated by acupuncture; ↓, downregulated by acupuncture. STHW, single-task habitual walking; DTHW, dual-task habitual walking; STFW, single-task fast walking; DTFW, dual-task fast walking; UPDRS, Unified Parkinson’s Disease Rating Scale; FES-I, short Falls Efficacy Scale-International; VAS, visual analog scale; HAM-A, Hamilton Anxiety Scale; PDQ-39, 39-item Parkinson Disease Questionnaire; NMSS, Non-Motor Symptoms Scale; ESS, Epworth Sleepiness Scale; PDSS, Parkinson Disease Sleep Scale; Hoehn and Yahr scale, HY scale; MMSE, Mini-mental State Examination; MFI-GF, Multidimensional Fatigue Inventory; GDS, Geriatrics Depression Scale; AES, Apathy Evaluation Scale; MAS, modified Ashworth score; KPPS, King’s Parkinson’s Pain Scale; VAS, visual analog scale; BSFS, Bristol Stool Function Scale; PAC-QOL, Patient Associated Constipation and Quality of Life Scale; BDI, Beck Depression Inventory; COGML/AP, medial-lateral (ML) center-of-gravity (COG) sway to anterior-posterior (AP) sway; ACTH, adrenocorticotropic hormone; CORT, cortisol; GV20, Baihui; GV14, Dazhui; LI4, Hegu; ST36, Zusanli; GB34, Yanglingquan; BL40, Weizhong; SP6, Sanyinjiao; KI3, Taixi; LR3, Taichong; GV24, Shenting; GV29, Yintang; HT7, Shen men; SP6, Sanyinjiao; BL62, ShenMai; KI6, ZhaoHai; TE5, Waiguan; PC6, Neiguan; CV6, Qihai; LU7, Lieque; SI19, Tinggong; LI15, Jianyu; LI11, Quchi; SP10, Xuehai; ST40, Fenglong; LR3, Taichong; CV4, Guanyuan; CV12, Zhongwan; ST25, Tianshu; GB20, Fengchi; LI15, Jianyu; LI 20, Yingxiang; ST7, Xiaguan; GV16, Fengfu; UB10, Tianzhu; GB12, Wangu; EX-B2, Jiaji.

### 3.1 Improving motor function

Multiple clinical studies have demonstrated that acupuncture can improve motor symptoms such as tremors, muscle rigidity, and bradykinesia in PD patients. Gait disorder is a key factor leading to falls and poor quality of life in PD patients. PD patients tend to walk on the ground with a low gait (high rhythm, short steps). Studies have objectively evaluated the efficacy of EA treatment for gait disorders in PD patients through body-worn sensor technology. After EA treatment, all gait parameters in the experimental group improved, including gait analysis during single-task habitual walking (STHW), dual-task habitual walking (DTHW), single-task fast walking (STFW), and dual-task fast walking (DTFW). In addition, the Unified Parkinson’s Disease Rating Scale (UPDRS), SF-12 health survey, short Falls Efficacy Scale-International (FES-I), and visual analog scale (VAS) scores have also been improved (Toosizadeh et al., 2015; Lei et al., 2016). Traditional MA treatment can also reduce UPDRS scores related to walking and balance, increase stride, swing, and single support time, and activate oxygenated hemoglobin in the cerebral cortex (Jang et al., 2020). PD patients may experience difficulty swallowing due to decreased muscle control ability. The swallowing process requires coordinated movement of multiple muscle groups. Acupuncture may help to improve the oral function of PD patients, by increasing the average tongue pressure and reducing the average latency of swallowing reflex, etc (Fukuda et al., 2016). Acupuncture can also effectively alleviate the pain of PD patients. For example, EA at *Baihui* (GV20), *Qihai* (CV6), *Lieque* (LU7), *Tinggong* (SI19), *Jianyu* (LI15), *Quchi* (LI11), *Xuehai* (SP10) and *Zusanli* (ST36) can reduce muscle hardness, effectively improve skeletal muscle pain s, enhance their daily living abilities, and also improve their depressive mood. The study also found that the degree of skeletal muscle pain is closely related to their motor ability and emotional disorders in PD patients (Shaosong et al., 2024). Fire Needling Therapy (FNT) is also one of the most common forms of acupuncture. It involves inserting a red-hot needle into the acupoints and causing a stronger response in the human body through additional heat stimulation, thereby achieving a better therapeutic effect. It can improve KPPS, VAS, UPDRS, and 39 item Parkinson’s Disease Questionnaire (PDQ-39) in PD patients, with significant differences observed in the 4th, 8th, and 12th weeks of treatment (Wang Y. et al., 2024).

### 3.2 Alleviating non-motor function

In addition to motor symptoms, acupuncture can also improve non-motor symptoms such as depression, anxiety, sleep disorders in PD patients. Anxiety is closely related to the accelerated progression of PD. After receiving 8 weeks of MA treatment, PD patients showed significant improvement in HAMA, UPDRS, and PDQ-39 scores, as well as downregulation of serum levels of adrenocorticotropic hormone (ACTH), thereby improving anxiety symptoms (Fan et al., 2022). The sleep disorders that occur in PD patients greatly affect their quality of life and accelerate the deterioration of the condition. Acupuncture also shows a positive effect on sleep disorders of patients with PD. In two randomized clinical trials (RCTs) in Brazil and China, acupuncture has been proved to be beneficial in improving the sleep quality and life quality of patients with PD, and significantly increase the Parkinson Disease Sleep Scale (PDSS). After follow-up, it was found that the therapeutic effect of acupuncture can even last up to 4 weeks (Aroxa et al., 2017; Yan et al., 2024). Fatigue is a common disability problem in PD patients, and there is currently no satisfactory treatment method. Preliminary studies have found that MA can improve symptoms in PD patients with moderate to severe fatigue, reduce the Multidimensional Fatigue Inventory (MFI-GF) and MFI-total score. Nevertheless, both real and sham acupuncture are equally effective in improving PD related fatigue, and further exploration is needed to determine whether this is a non-specific or placebo effect (Kluger et al., 2016; Kong et al., 2018). Eight weeks of scalp-abdominal EA treatment can effectively improve the NMSS, PDSS, and UPDRS scores of PD patients. Both sub domains of NMSS, such as sleep/fatigue and miscellaneous, have significant effects. However, although the scalp-abdominal EA treatment is targeted at the intestinal tract, it has no effect on Bristol Stool Function Scale (BSFS) and Patient Associated Constipation and Quality of Life Scale (PAC-QOL). After acupuncture intervention, the relative abundance of genera Bacteroides and Parasutterella is increased, while the abundance of genera Dialister, Hungatella, Barnesiella, Megasphaera, Allisonella, Intestinimon, and Moryella is significantly decreased. However, regardless, microbiota-gut-brain-axis (MGBA) may be one of the possible mechanisms for acupuncture treatment of PD (Nazarova et al., 2022). Acupuncture combined with other therapies also has a good effect in the treatment of PD. A study has observed the therapeutic effect of acupuncture combined with tuina and instrument-delivered qigong on idiopathic PD patients and found that the treatment plan is safe and highly accepted for PD patients. Most subjective symptoms have improved, such as Beck Depression Inventory (BDI) and PDQ-39, but it has no significant effect on UPDRS motor score (Eng et al., 2006).

## 4 Acupuncture alleviate PD via GABAergic signaling

The pathological process of PD has not been fully elucidated. However, numerous basic studies have shown that acupuncture improves motor and non-motor symptoms of PD patients. Functional magnetic resonance imaging (fMRI) shows that when PD patients receive acupuncture treatment, the putamen and primary motor cortex are activated, and the expectation of acupuncture methods trigger activation of the anterior cingulate gyrus, superior frontal gyrus, and superior temporal gyrus, which are associated with enhanced individual motor function. The basal ganglia-thalamocortical circuit may be an important link in promoting the improvement of motor function by acupuncture in PD patients (Chae et al., 2009). There are abundant GABAergic neurons in the basal ganglia-thalamocortical circuit and considering the important role of GABAergic signaling in the pathogenesis and progression of PD, we hypothesize that acupuncture may have a therapeutic effect on PD by regulating GABAergic signaling. And this review describes how acupuncture helps alleviate PD-related symptoms via GABAergic signaling.

Under normal physiological conditions, GABA acts as an inhibitory neurotransmitter and participates in regulating the excitability of motor neurons. Acupuncture can regulate the synthesis and release of GABA, as well as the expression and activity of GABA receptors, thereby treating diseases. For example, acupuncture can enhance the expression of GABA, potassium-chloride cotransporter 2, and GABA_Aγ 2_ in the brainstem of rats, thereby improving the structure of spastic muscles and decreasing spasticity (Sun et al., 2022). After 1 week of acupuncture at the *Jiaji* (EX-B2) acupoints, the expression of GABA_Aγ 2_ and GABA_BR2_ in the striatum and spinal cord were significantly increased, thereby improving locomotor function of rats (Xu et al., 2015). For patients with insomnia, it has been reported that acupuncture at GV20, GV29, GV24 can improve sleep quality, reduce falling asleep time, increase sleep time, improve sleep efficiency, and lower sleep disturbance scores, which may be related to the increasing serum GABA levels and inhibition of the hypothalamic pituitary adrenal (HPA) axis. The therapeutic effect of acupuncture is almost the same as that of western medication estazolam tablets, but with fewer side effects (Wu et al., 2021). Acupuncture can also effectively upregulate GABA and GABA_A_ receptors in the hypothalamus of rats caused by insomnia, prolong pole-climbing time, and help alleviate insomnia. This study also compared the therapeutic effects of acupuncture at different acupoints and found that acupuncture at *Shenmen* (HT7) and *Shenmai* (BL62)-*Zhaohai* (KI6) was superior to acupuncture at PC6, ST36 and *Sanyinjiao* (SP6) in insomnia rats (Zhou et al., 2012; Kalia and Lang, 2015).

When rats experience pain and anxiety, there is an increase in GABAergic neurons in the midcingulate cortex (MCC). Research has found that different GABA receptors play different roles, with GABA_B_ receptor inhibition playing a key role in pain memory and anxiety-like behavior, while GABA_A_ receptor is not involved in this mechanism. EA at ST36 can reverse pain and anxiety-like behavior in rats by inhibiting GABA_B_ receptor in MCC (Li X. et al., 2023). However, in the anxiety-like behavior caused by morphine withdrawal treatment, acupuncture at HT7 alleviates this anxiety-like behavior through the mediation of the GABA_A_ receptor system (Kim et al., 2018). This suggests that the mechanism of acupuncture regulating the GABA receptor system in treating PD still needs further exploration. EA at ST36 and SP6 can upregulate hippocampal metabolites, mainly containing l-glutamine and GABA, further blocking the TLR4/nuclear factor kappa B (NF-κB) signaling pathway and NLRP3 inflammasomes, while downregulating IL-1β levels, inhibiting excessive activity of the HPA axis, and attenuating anxiety and depression like behavior in rats (Zhou et al., 2022). The rostral anterior cingulate cortex (rACC) is a critical region for regulating harmful behavior and negative emotions. EA at ST36 can also alleviate pain and anxiety-like behaviors associated with pain memory by activating GABAergic neurons and GABA_A_ and GABA_B_ receptors in rACC of memory model rats (Sun et al., 2024).

The medulla oblongata is an important hub connecting the brain and spinal cord, responsible for transmitting neural signals. The nucleus tractus solitarius (NTS) is a neural nucleus located in the medulla oblongata. The starting nucleus of the vagus nerve is also located in the medulla oblongata. There are abundant neural connections between the dorsal motor nucleus of vagus nerve (DMV) and the NTS. Research has found that EA can significantly activate NTS neurons, further drive vagus nerve output by inhibiting the expression of GABA_A_ receptor in DMV, thereby alleviating inflammation (Yang et al., 2021). In 6-OHDA induced PD rats, EA at GV20 and *Dazhui* (GV14) can effectively improve their abnormal rotational behavior, decrease the ratio of GABA content on the lesioned side to that on unlesioned side in the cortex, and increased the ratios in the striatum and cerebellum. The effect of high-frequency EA may be exerted by enhancing the inhibitory effect of the cerebellum-basal ganglia-cortical loop on the motor center (Du et al., 2011).

The neuroplasticity of GABAergic neurons in the BG of PD patients is reduced, leading to impaired neuronal function. Acupuncture may regulate the neuroplasticity of GABAergic neurons, regulate synaptic transmission, modulate the inhibitory effects of GABA neurons, and improve the function of GABAergic neurons. EA at *Hegu* (LI4) and *Taichong* (LR3) can regulate the content of neurotransmitters (upregulating 5-HT and GABA, downregulating Glu levels), upregulate the expression of synapse-associated proteins such as Syn and PSD-95, activate the brain-derived neurotrophic factor (BDNF)/tyrosine-protein kinase B (TrKB)/cAMP response element binding protein (CREB) signaling pathways, ameliorate morphological abnormalities and preserve synaptic ultrastructure of neural cells in the hippocampus of rats, finally alleviate spinal hyperreflexia and motor dysfunction (Yang P. et al., 2023). Acupuncture at *Shuigou* (GV26) can effectively reduce ischemia-induced excessive release of Glu and maintain the endogenous inhibitory activity of GABA. It was found that this effect occurs throughout the acupuncture treatment and lasts for a period of time after the end of acupuncture (Gan et al., 2005; Liu et al., 2019). In the rat PD model induced by medial forebrain bundle (MFB) transection, it was found that high-frequency EA (100 Hz) at the GV14 and GV20 acupoints can reduce rotational behavior, improve motor coordination, and protect dopaminergic neurons with axonal transection from degeneration of the SN, but it does not increase striatal DA levels. However, EA significantly decreases the levels of Glu and ACh in the damaged side of the striatum. This suggests that EA treatment for PD involves a non-dopamine pathway, and Glu is a precursor to GABA synthesis. The interaction between Glu and GABA in the nervous system maintains the balance of neural activity, which further suggests that GABAergic signals may play a role in EA treatment (Sun et al., 2012).

## 5 Conclusion

PD, as a common neurodegenerative disorder, severely affects the quality of life of patients. GABA is an important inhibitory neurotransmitter in the central nervous system, acting by binding to specific receptors (GABA_A_/GABA_B_). The GABAergic signaling pathway plays a crucial role in the occurrence and development of PD, involving motor symptoms as well as non-motor symptoms such as mood disorders and sleep disorders. Acupuncture, as a traditional Chinese medicine therapy, has shown certain potential in the treatment of PD. Acupuncture can ameliorate motor and non-motor symptoms in PD patients, and its mechanism may be related to regulating the function of GABAergic neurons. However, the current evidence mainly comes from related studies, and future research needs to further explore the mechanism of acupuncture in regulating GABAergic signaling in the treatment of PD, optimize acupuncture treatment plans, and improve the efficacy of acupuncture treatment. At the same time, multi center and large-scale clinical studies are required to provide stronger evidence for acupuncture treatment of PD.
